# Cerebral cortical processing time is elongated in human brain evolution

**DOI:** 10.1038/s41598-022-05053-w

**Published:** 2022-01-20

**Authors:** Kosuke Itoh, Naho Konoike, Masafumi Nejime, Haruhiko Iwaoki, Hironaka Igarashi, Satoshi Hirata, Katsuki Nakamura

**Affiliations:** 1 Center for Integrated Human Brain Science, Brain Research Institute, Niigata University, Niigata, Japan; 2 Cognitive Neuroscience Section, Primate Research Institute, Kyoto University, Inuyama, Aichi 951-8585 Japan; 3 Wildlife Research Center, Kyoto University, Kyoto, Japan; 4 Present Address: Division of Biomedical Science, Faculty of Medicine, University of Tsukuba, Tsukuba, Japan

**Keywords:** Auditory system, Cognitive neuroscience, Neurophysiology

## Abstract

An increase in number of neurons is presumed to underlie the enhancement of cognitive abilities in brain evolution. The evolution of human cognition is then expected to have accompanied a prolongation of net neural-processing time due to the accumulation of processing time of individual neurons over an expanded number of neurons. Here, we confirmed this prediction and quantified the amount of prolongation in vivo, using noninvasive measurements of brain responses to sounds in unanesthetized human and nonhuman primates. Latencies of the N1 component of auditory-evoked potentials recorded from the scalp were approximately 40, 50, 60, and 100 ms for the common marmoset, rhesus monkey, chimpanzee, and human, respectively. Importantly, the prominent increase in human N1 latency could not be explained by the physical lengthening of the auditory pathway, and therefore reflected an extended dwell time for auditory cortical processing. A longer time window for auditory cortical processing is advantageous for analyzing time-varying acoustic stimuli, such as those important for speech perception. A novel hypothesis concerning human brain evolution then emerges: the increase in cortical neuronal number widened the timescale of sensory cortical processing, the benefits of which outweighed the disadvantage of slow cognition and reaction.

## Introduction

“Bigger is better” is a simple and widely accepted principle in brain evolution. The uniquely developed cognitive abilities of humans, such as language and abstract thinking, are supported by the disproportionate increase in the human brain size, which occurred to an extent that surpasses the general allometric relationship between body size and brain size in mammals^1,2^. An important assumption behind this hypothesis is that a larger brain contains a greater number of neurons, particularly cortical neurons, which augments the capacity for complex information processing. Although recent studies have shown that brain size is not a reliable predictor of the number of cortical neurons when compared across clade^3^, cerebral neuronal number increases with brain size within a clade, and primates have the highest density of cortical neurons among other mammals of similar brain size^4^. Consequently, the evolutionarily scaled-up human brain contains a greater number of cerebral cortical neurons than the African elephant, which has a brain three times larger than that of an average human^5^.

However, there is a sometimes overlooked disadvantage to having an increased number of cortical neurons, namely, a prolonged neural-processing time. An increase in the total number of cortical neurons implies that the number of neurons that support a specific cortical function, e.g., detecting a sound event or identifying a familiar face, also increases. Then, the total neural-processing time for executing a brain function is expected to become longer unless (1) all evolutionarily acquired cortical neurons operate in parallel with previously existing neural circuits, or unless (2) the temporal integration time of individual neurons (i.e., the time required by a neuron to summate synaptic inputs before it elicits an action potential) is shortened with the increase in the total neuronal number. To the best of our knowledge, there is no evidence to support these assumptions.

Some mechanisms have been hypothesized to mitigate the expected lengthening of the neural-processing time in the human brain, namely, cortical folding^1,6^, high neuronal density^4^, and thickening of myelination^7^ that would contribute to maintaining a short axonal conduction time. However, to the best of our knowledge, these hypotheses have not been tested in vivo because of the difficulty to measure the speed of neural processing in the cortical tissues of living humans and nonhuman primates.

Did the human brain trade off neural-processing speed for the benefit of elaborate computation? To address this problem, we used scalp electroencephalogram (EEG) to measure the speed at which neural circuits in the cerebral cortex process sensory information in four living primates: the human (*Homo sapiens*), chimpanzee (*Pan troglodytes*), rhesus monkey (*Macaca mulatta*), and common marmoset (*Callithrix jacchus*). These species have vastly different brain sizes and hence considerably different number of neurons (Table 1). Scalp EEG has recently been applied to nonhuman primates as a novel and powerful tool in comparative neurophysiology, as it reveals species differences in neural processing that underpin the evolution of cognition and behavior^8–14^.

**Table 1:** Brain size and number of neurons in the four primate species investigated in this study.

Species	Brain mass (g)^34^	Number of cortical neurons^36^	Estimated number of auditory cortical neurons^a^
Common marmoset	7.64	244,720,000	1,713,04
Rhesus monkey	88.94	1,710,000,000	11,970,000
Chimpanzee	376.13	4,900,821,995*	31,933,022
Human	1250.43	16,340,000,000	102,678,300

*Predicted from brain mass by using equation in^57^.

^a^See “Supplementary analysis of allometry” in the “Results” section for the estimation method.

This study focused on audition, which represents one of the most time-critical sensory modalities in the brain: sub-second temporal processing of sounds is fundamental to virtually all forms of audition, including speech perception. Specifically, the peak latencies of the P1 and N1 components of the cortical auditory evoked potential (CAEP) were exploited as a neurophysiological measure of neural processing time in the auditory cortex. EEG signals reflect the sum of postsynaptic potentials of many neurons that are activated in synchrony. Therefore, the peak latency of P1 or N1 indicates the timepoint at which a specific group of neurons subserving a particular stage of auditory processing were activated together in a functionally connected network.

A major generator of the human P1, which peaks around 50 ms after sound onset, is located in the primary auditory area^15^. Considering that single neurons in the human auditory core fire as early as 10–20 ms in latency following auditory stimulus^16,17^, the delayed latency of P1 at approximately 50 ms provides evidence of the first stages of neuronal processing in the neocortical circuit. Such delay appears to be a common property of the mammalian auditory cortex, as epicranial CAEP in mice indicated auditory cortical activities between 18 and 25 ms and between 25 and 54 ms in latency^18^, although intracranial recordings revealed that neural activities in the same cortical region begin earlier at approximately10 ms^18,19^.

After approximately 50 ms following P1, further cortical processing culminates in N1, which is hypothesized to be generated in the primary auditory cortex as well as in other cortical areas^20^. N1 reflects a higher level of auditory processing than P1 and marks the beginning of a sound event as segmented in the auditory cortex^20,21^. Segmentation is based on the detection of abrupt changes in acoustic features, such as intensity and spectrum^20,22,23^, as well as higher-order changes in spectrotemporal patterns^24^. Such segmentations require analyzing sound inputs over a time window that is sufficiently long to embrace the acoustic change.

Importantly, despite the large differences in brain size, there are only a few milliseconds of difference in the response latencies of brainstem and midbrain neurons between humans, apes, and monkeys, as reflected by waves I (latency: 1–2 ms) through V (latency: 5–7 ms) of auditory brainstem responses^25–28^. Moreover, invasive intracranial recordings have revealed that the minimum response latencies of primary auditory cortical neurons are strikingly similar across primates: these are approximately 10–20 ms in humans^16,17^, macaques^29,30^, and marmosets^31,32^ alike, although there are variations depending on experimental conditions. Accordingly, any differences in P1 and N1 latency between species would represent differences in auditory cortical processing time, rather than differences in neural conduction time from the cochlea to the cortex.

## Results

### Main

**Figure 1:**
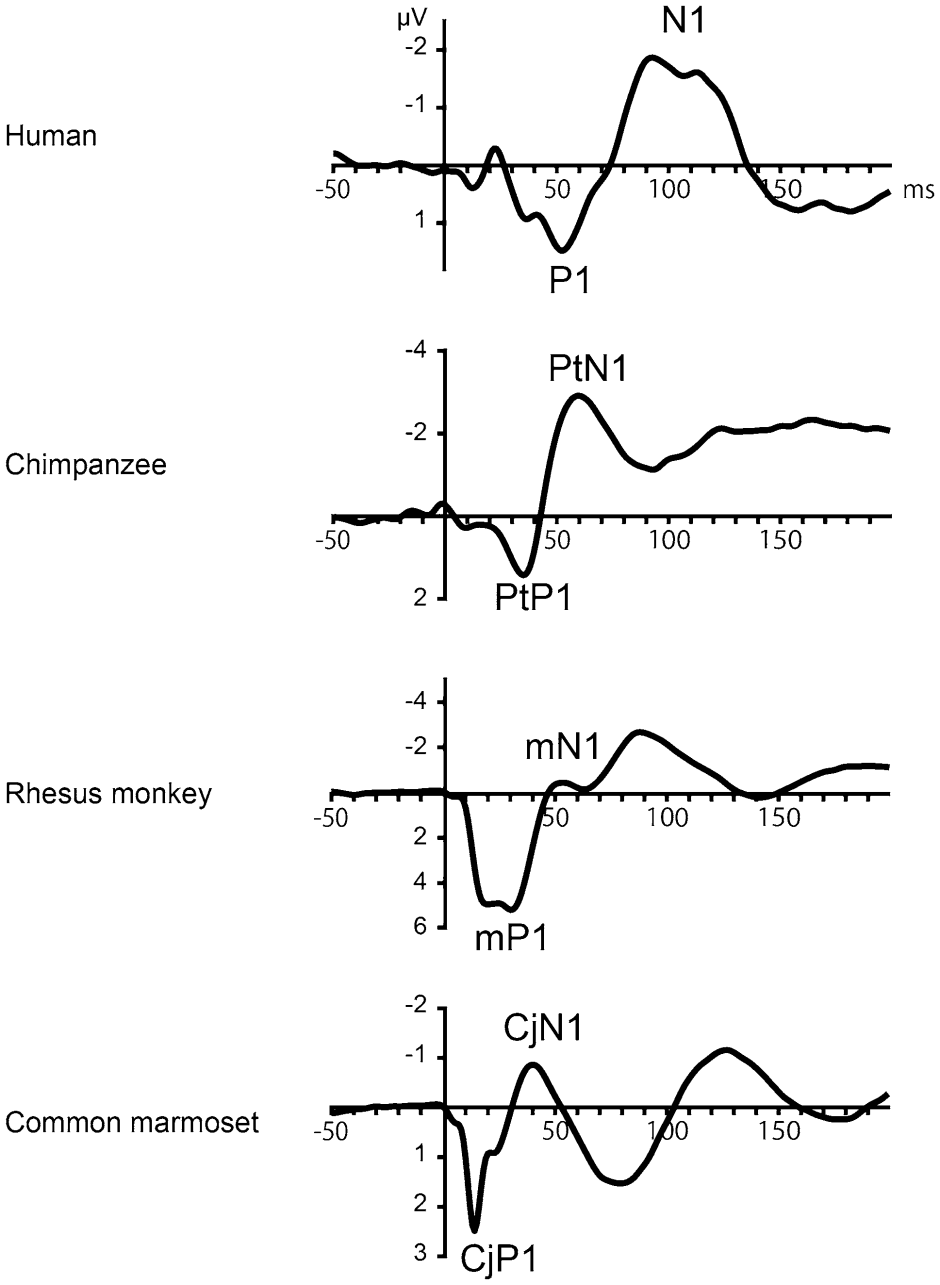
Scalp-recorded CAEPs in the four primate species investigated in the study. The homologs of human P1/N1 were identified as PtP1/PtN1 in the chimpanzee, mP1/mN1 in the rhesus monkey, and CjP1/CjN1 in the common marmoset. The latencies of P1/N1 homologs are elongated as the brain size increases. The electrode was placed at the vertex. *CAEP* cortical auditory-evoked potential.

We measured the latencies of P1 and N1 in humans (*n* = 18), a chimpanzee (*n* = 1), rhesus monkeys (*n* = 7), and marmosets (*n* = 4) to investigate this issue. The measurements of P1 and N1 latencies were obtained by reanalyzing the raw data from published and unpublished experiments by our group^9,11–14^, which were selected so that the stimuli and recording conditions were matched as closely as possible between the species. All recordings were made in a passive listening condition without a behavioral task in an awake state, to ensure that the CAEPs reflected exogenous and automatic response to sounds with minimal confounding effects of attention and other higher functions. The obtained CAEP waveforms in all species shared the same feature that the first prominent peak was positive in polarity, representing the P1 and, naturally, the negative peak that immediately followed it represented the N1 (Fig. 1). The homologs of the human P1 and N1 have been identified in the rhesus monkey (labeled mP1 and mN1, “m” standing for macaque)^12,13^ and in the common marmoset^11^ (CjP1 and CjN1, “Cj” standing for *Callithrix jacchus*). Precisely, the CjP1 is a composite of two positive waves, CjPa and CjPb, and the detailed analyses of these subcomponents are presented in the next section (Supplementary analysis of P1); these yielded consistent results. This is the first study to identify the homologs of P1 and N1 in the chimpanzee, which were labeled here as PtP1 and PtN1, respectively, where “Pt” stands for *Pan troglodytes*.

**Figure 2:**
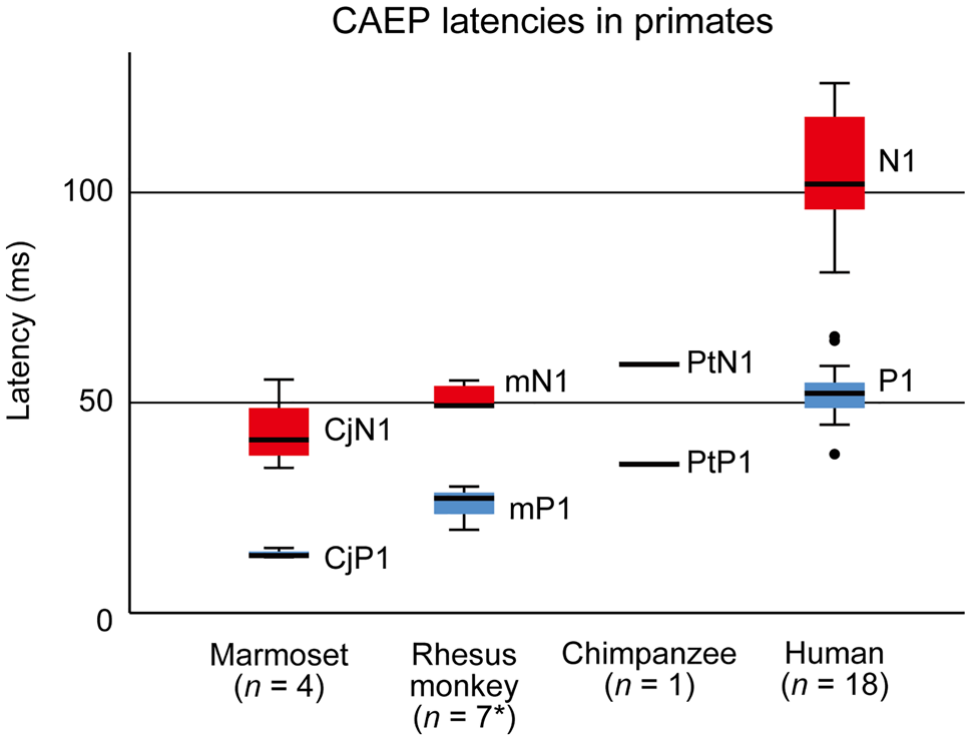
Box plot of the latencies of P1/N1 homologs in the four primate species. The thick horizontal line indicates the median, and the top and bottom box lines show the first and third quartiles. The whiskers show the maximum and minimum values, excluding outliers (circles) that were at least 1.5 box length from the median. Numbers in parentheses below the species names indicate the number of subjects (*n*). *Two rhesus monkeys were excluded for the evaluation of mN1, as the peak was not clearly identified in the individual CAEP waveforms. The mean ± standard deviation of the peak latencies was as follows: 14 ± 1 (CjP), 26 ± 4 (mP1), 36 ± 3 (PtP1), and 53 ± 7 (P1) for the P1 homologs; 43 ± 9 (CjN1), 51 ± 3 (mN1), 59 ± 3 (PtN1), and 104 ± 15 (N1) for the N1 homologs. The standard deviations were calculated over subjects in the common marmosets, rhesus monkeys, and humans, and over experimental conditions and sessions in the chimpanzee.

The peak latencies of P1 and N1 homologs increased as the brain enlarged in size (Figs. 1 and 2), and the differences between the species were statistically significant for P1 (Kruskal–Wallis *H* = 22.4, *P* = 0.0001) and N1 (*H* = 19.4, *P* = 0.0003). As the latencies of auditory brainstem responses have been relatively preserved in primate evolution^25–28^, the lengthening of the P1 and N1 latencies indicated an extension of the dwell time of cortical processing that cooccurred with brain enlargement. A possible anatomical basis for the selective delay of higher cortical processing, compared to lower subcortical processing, is a disproportionate increase in the number of auditory cortical neurons. In the primate auditory system, the number of neurons in the auditory cortex and subcortical structures (i.e., inferior colliculus and medial geniculate nucleus) both increase with brain size, but the rate of the increase is greater in the former^33^; hence, larger primate brains have increased ratios of cortical to subcortical neurons that are involved in processing sounds. Accordingly, the observed lengthening of the P1 and N1 latencies is consistent with the hypothesis that neural-processing time increases with the accumulation of the number of neurons leading to the production of the neural response.

To further investigate this hypothesis, we studied the allometric relationships between the P1/N1 latencies and two metrics of brain size, namely, the number of cortical neurons *N* as reported in the literature, and the equivalent brain radius *R*_eq_ (mm). For an alternative analysis using estimated numbers of auditory cortical neurons, *N*_auditory_, instead of *N*, please refer to the section below: Supplementary analysis of allometry. The equivalent brain radius *R*_eq_ was obtained by solving the equation, $V=\frac{3}{4}\pi {{R}_{\text{eq}}}^{3}$, where the brain volume *V* was calculated by dividing the brain mass (g) as reported in the literature (Table 1)^34^ by the specific gravity of the brain tissue, 1.036 g/cm^3^^35^. As the auditory pathway length would scale with the cube root of brain volume, the CAEP latencies would increase linearly with *R*_eq_, if these mostly reflected the conduction time of neural signals along axons in subcortical and cortical structures. Alternatively, latencies would scale linearly with *N* if these mostly reflected the sum of processing time of individual neurons, as each neuron requires time to summate excitatory and inhibitory synaptic inputs before it elicits an action potential, and this delay would accumulate with increasing *N*.

**Figure 3:**
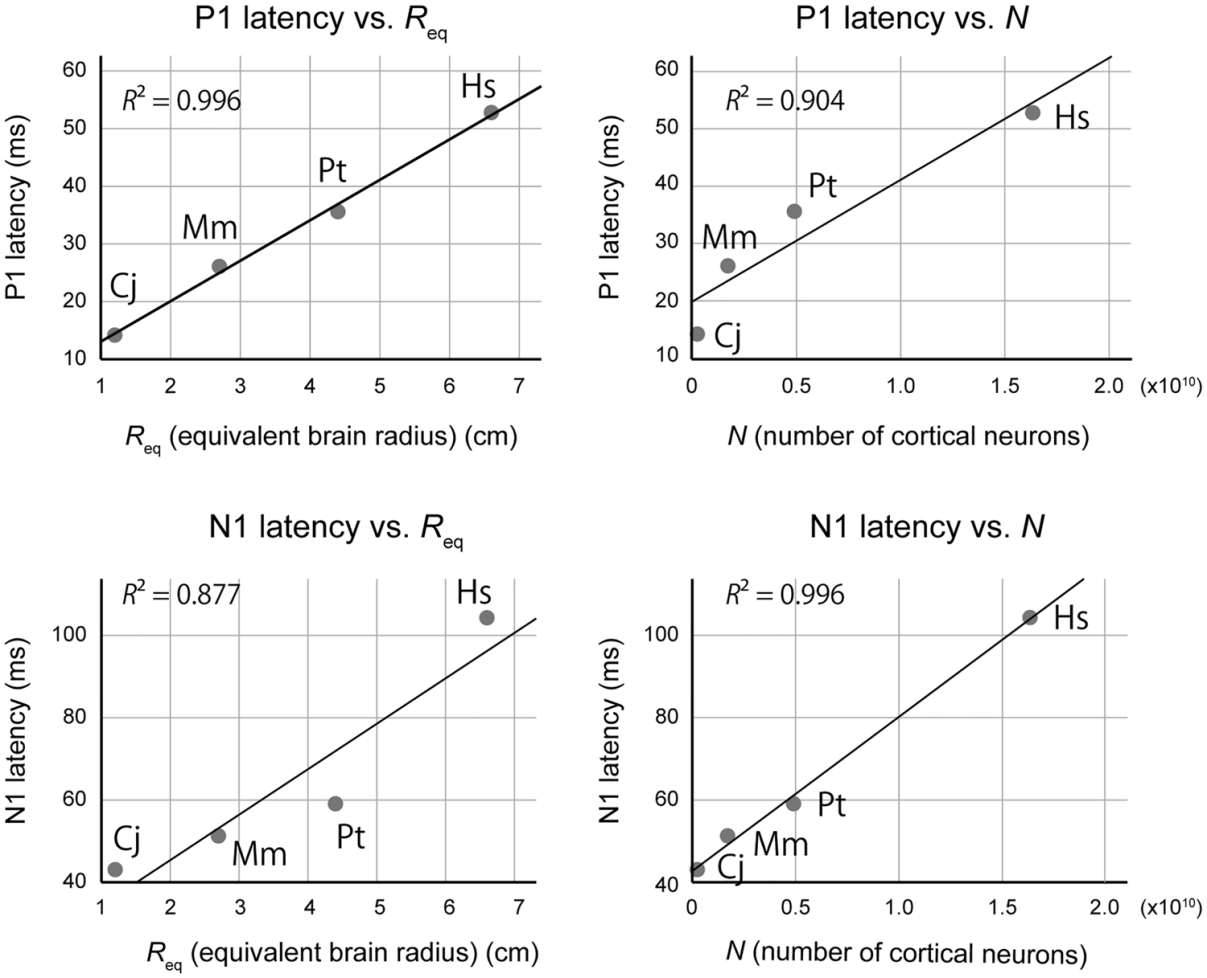
Allometry of P1/N1 latencies with respect to brain size (equivalent brain radius, *R*_eq_ cm) and the number of neurons in the brain (*N*). The P1 latency showed a higher correlation with *R*_eq_ than with *N*, while the N1 latency showed a higher correlation with *N* than with *R*_eq_. *Cj* common marmoset, *Mm* rhesus monkey, *Pt* chimpanzee, *Hs* human.

The results supported the latter hypothesis regarding N1, as *N* was a better predictor of its latency (*R*^2^ = 0.996, *P* = 0.002) than *R*_eq_ (*R*^2^ = 0.877, *P* = 0.063) (Fig. 3). However, the opposite results were obtained for P1, as the effect size was greater with *R*_eq_ (*R*^2^ = 0.996, *P* = 0.002) than with *N* (*R*^2^ = 0.904, *P* = 0.049). Thus, although the number of neurons and physical brain size both contributed to prolonging the CAEP latency, the former factor became more dominant at a higher level of cortical processing, as manifested by the prominent delay of human N1 latency (Fig. 2).

### Supplementary analysis of P1

**Figure 4:**
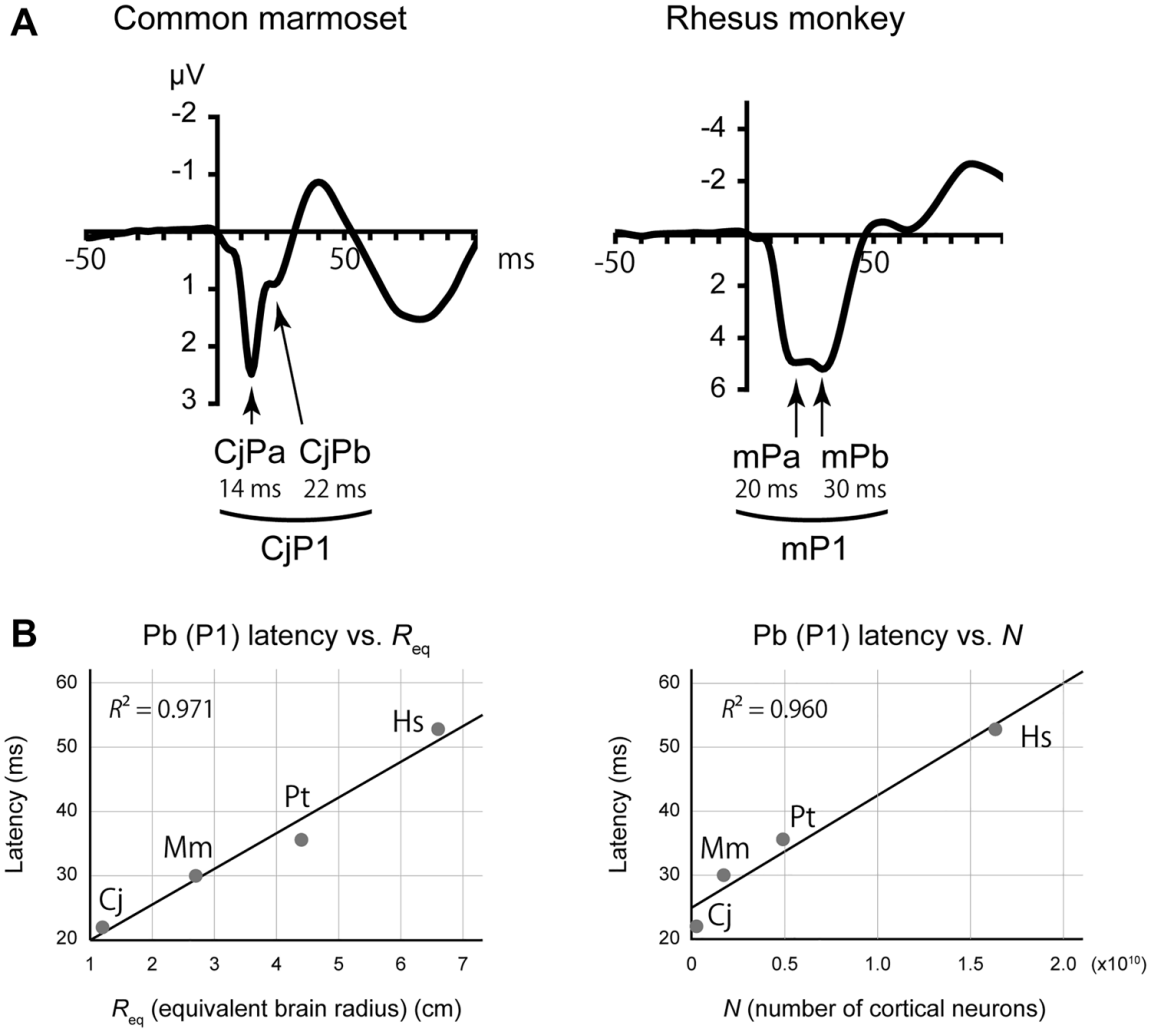
Detailed waveforms of the putative P1 homologs. (**A**) The marmoset CjP1 and rhesus mP1 comprised two subcomponents that putatively corresponded to the human MLR components Pa and Pb. (**B**) Allometry of P1 latencies with respect to brain size (equivalent brain radius, *R*_eq_ cm) and the number of neurons in the brain (*N*), in which the subcomponents of CjP1 and mP1 were distinguished. The results were similar to those of the original analysis (Fig. 1, main text). MLR, middle latency response. *Cj* common marmoset, *Mm* rhesus monkey, *Pt* chimpanzee, *Hs* human.

A careful inspection of the CAEP waveforms in Fig. 1 suggested that the CjP1 in the marmoset comprised two subcomponents that were not clearly resolved in time (Fig. 4A). In fact, our previous analysis of the high frequency components of marmoset auditory-evoked potential (AEP) revealed that CjP1 is a complex of two middle latency responses (MLRs), CjPa and CjPb, which putatively correspond to the human MLR components Pa and Pb, respectively^11^. MLRs are AEPs that occur after brainstem responses (< 10 ms in humans) and before the CAEP (> 50 ms in humans); they can be recorded using a higher EEG sampling rate and a higher passband frequency than those used for CAEPs. In the present study, the Pa and Pb were lumped together to form a single positive peak P1, owing to the low-pass filter that we used to focus on the late cortical components.

According to widely accepted nomenclature, the human MLR component Pb is equivalent to the human CAEP component P1. However, in the marmoset MLR, CjP1 (which is a lowpass-filtered CjPa–CjNa–CjPb complex) was dominated by CjPa, because it was much larger in amplitude than the CjPb (Fig. 4A), as has been previously described in detail^11^. Accordingly, there was a concern that our analysis of CjP1 latency reflected the latency of CjPa rather than that of CjPb, although it is the latter that represents the putative homolog of the human P1.

The situation was somewhat similar in macaques. Although, to our knowledge, the component structure of the rhesus monkey scalp MLR has not been clarified, the waveforms in the present study suggested that it also comprised two positive peaks: mPa and mPb. Assuming that mPa and mPb are homologs of CjPa and CjPb, respectively, then the second subcomponent mPb represents the candidate homolog of human P1. Nevertheless, as mPa and mPb were comparable in amplitude (Fig. 4A), our measurement of the mP1 latency in the above analysis probably did not accurately represent mPb latency.

By contrast, the PtP1 in the chimpanzee was similar in waveform to human P1, in that it only had one clear peak. Therefore, we could tentatively assume, although without concrete evidence, that PtP1 corresponded to human Pb (or P1) rather than to human Pa.

Table 2 summarizes the proposed correspondences of P1 and its subcomponents between species, as described above.

**Table 2:** Putative homologies of Pa/Pb (MLR) and their relationship with P1 (CAEP).

	Human		Chimpanzee		Rhesus monkey		Common marmoset	
	MLR	CAEP	MLR	CAEP	MLR	CAEP	MLR	CAEP^a^
Putative homologs	Pa	Small, usually not seen	n.a	Was not seen	mPa	First peak of mP1	CjPa	Main peak of CjP1
Putative homologs	Pb	P1	n.a	PtP1	mPb	Second peak of mP1	CjPb	Small hump in CjP1

*n.a.* information not available, to our knowledge. *CAEP* cortical auditory-evoked potential, *MLR* middle latency response.

^a^The first positive CAEP component, referred to here as CjP1, was denoted as CjP1 in our previous study to distinguish it from the “true” CjP1, or CjPb.

To take into account these considerations, the allometric analyses in the main analyses (Fig. 3) were repeated by using the latencies of CjPb and mPb, rather than those of CjP1 and mP1, to aim at a more accurate cross-species correspondence of components. The latencies of CjPb and mPb were obtained by visually inspecting the grand-average waveforms (Fig. 4A, marked with arrows).

As a result, the findings were similar to those of the original analyses of P1 homologs. That is, the effect size was greater for the equivalent brain radius *R*_eq_ (*R*^2^ = 0.971, *P* = 0.015) than for the number of cortical neurons *N* (*R*^2^ = 0.960, *P* = 0.020) (Fig. 4B), although the effect of *N* was slightly greater (and that of *R*_eq_ was slightly smaller) than that of the original analysis.

### Supplementary analysis of allometry

In the main analysis above, elongations of P1 and N1 latencies were analyzed with respect to differences between species in the total number of cortical neurons *N*. However, since the P1 and N1were auditory cortical responses, the number of auditory cortical neurons *N*_auditory_ may be a more appropriate independent variable.

Regarding common marmosets and rhesus monkeys, the size of auditory cortex scales proportionally with that of the entire cerebral cortex, maintaining a relative number of neurons of 0.7%^33^. Therefore, the *N*_auditory_ in these species could be estimated from the *N*s reported in the literature^36^ as listed in Table 1, referred to as Estimation 1.

However, the proportion *N*_auditory_/*N* was expected to be smaller than 0.7% in the chimpanzee and the human, which have an evolutionarily well-developed prefrontal cortex. Therefore, the number of auditory cortical neurons in these species were estimated by a different method. With *P* as the relative volume of the prefrontal cortex, the relative volume of “the rest of the cortex” would be 1 − *P*. Assuming that a fixed proportion, *p*, of “the rest of the cortex” is occupied by the auditory area in all four species, the relative volume of the auditory cortex with respect to the whole cortex would be (1 − *P*) * *p*. Here, *P* has been measured to be 0.377, 0.354, and 0.306 for the human, chimpanzee, and macaque, respectively^37^. Although the measurement was not available for the common marmoset, we considered this be identical to that of the macaque. A small error in this estimation would only have an insignificant effect on the results, as discussed below.

By using these values of *P*, the number of auditory cortical neuron *N*_auditory_ was estimated as 0.694 * *p* * *N*_marmoset_ for marmosets, 0.694 * *p* * *N*_macaque_ for macaques, 0.646 * *p* * *N*_Chimpanzee_ for chimpanzees, and 0.623 * *p* * *N*_human_ for humans, where *N*_species_ was the total number of cortical neurons in that species: we refer to these as Estimation 2. Here, by equating Estimations 1 and 2 for the marmoset and the macaque, *p* was calculated as 0.010, and *N*_auditory_ in the human and the chimpanzee were obtained by inserting this value of *p* in Estimation 2, as shown in Table 1.

The analysis of allometry was performed with these estimates of *N*_*auditory*_. As a result, a higher correlation was observed between the N1 latency and *N*_*auditory*_ (*R*^2^ = 0.996, *P* = 0.002) than between *N*_*auditory*_ and P1 (*R*^2^ = 0.918, *P* = 0.042). These results closely replicated the findings of the main analysis above. The robustness of this finding was anticipated because in the scope of a comparative study that spans several orders of magnitude, differences between *N* and *N*_*auditory*_ are minor. For the same reason, small variations in *P* or *p* would only have an insignificant effect.

## Discussion

An elongation of the auditory cortical processing time entails both merits and demerits. Slow processing is disadvantageous in situations where fast identifications of sounds are required, such as when detecting the sound of a predator. On the other hand, a widened processing time has the advantage of allowing the auditory input to be integrated over a longer time window^13^, which is beneficial for analyzing the time-varying temporal structures of sounds, such as those important for the segmentation and categorization of phonemes. It may be that the evolution of speech in humans was supported by the enhanced capacity for sound perception that was made possible by the longer cortical processing time.

In fact, the hypothesis that the auditory cortical time window elongated in humans explains many of the previously reported differences between species in primate sound perception, particularly regarding those involving the processing of time. Discrimination of sound duration is more difficult in monkeys than in humans^38^, and detectability of small frequency changes improved with stimulus duration more in humans than in monkeys^39^. Monkeys are less sensitive than humans to temporally global features of spectrotemporal variation in sounds and focus on local features in cognitive tasks^40^. The cutoff frequency of the temporal modulation transfer function, which reflects the low-pass filter characteristic of an auditory system, is lower in humans than in macaques^41^. These diverse species differences in sound perception are consistent with a longer auditory cortical time window in humans.

Although our findings highlighted the impact of increased neuronal number in human brain evolution, we also acknowledge the importance of brain size enlargement. For example, the long-range neural communication between the two cerebral hemispheres is constrained in speed as brain size increases, because of the limit of the action potential propagation speed^42^. Such constraint has been proposed to have promoted the evolution of hemispheric specialization in enlarged brains, so that fast, time-critical computations are performed by short and small circuits in one hemisphere^43,44^. However, brain enlargement would also increase transmission delays in long-range intra-hemispheric connections such as the arcuate fasciculus, and communications via these fibers may be slower than those across the commissure between homologous cortical areas. Thus, neuronal number and brain size have complicated effects on the evolution of neural circuitries that support human cognition, the details of which remain to be elucidated.

Although the neural generators of the P1s and N1s investigated in this study were mostly unknown, a putative macaque homolog of P1 at 28 ms was generated by current sources located in lamina II of the primary auditory cortex, which were balanced by sinks located in upper lamina III^45^. This sink-source configuration was consistent with polysynaptic activation of supragranular pyramidal cell elements that followed the earliest current sinks that occurred within the thalamorecipient zone (layer IV and III) at a latency of 17 ms^45^. Subsequently, a putative macaque homolog of N1 at around 50 ms was coincident in time with a superficial sink in the supragranular layer of the primary auditory cortex, balanced by a deeper source^45,46^. Therefore, assuming that our proposed homologies were valid, the CAEP components analyzed in this study likely reflected initial stages of the neural processing of sounds in the primary auditory cortex, although sources outside the primary auditory cortex may also have contributed to the CAEPs. As further evidence, all P1s and N1s were recorded maximally around the vertex, and this scalp topography was consistent with localization of the sources on the superior temporal plane within the lateral sulcus. Although there are anatomical variations across and within species, most of the primary auditory cortex is located on the upper bank of the lateral sulcus in humans, chimpanzees, and macaques^47^, whereas it extends laterally from within the lateral sulcus onto the lateral surface of the superior temporal gyrus in the marmoset^48^.

**Figure 5:**
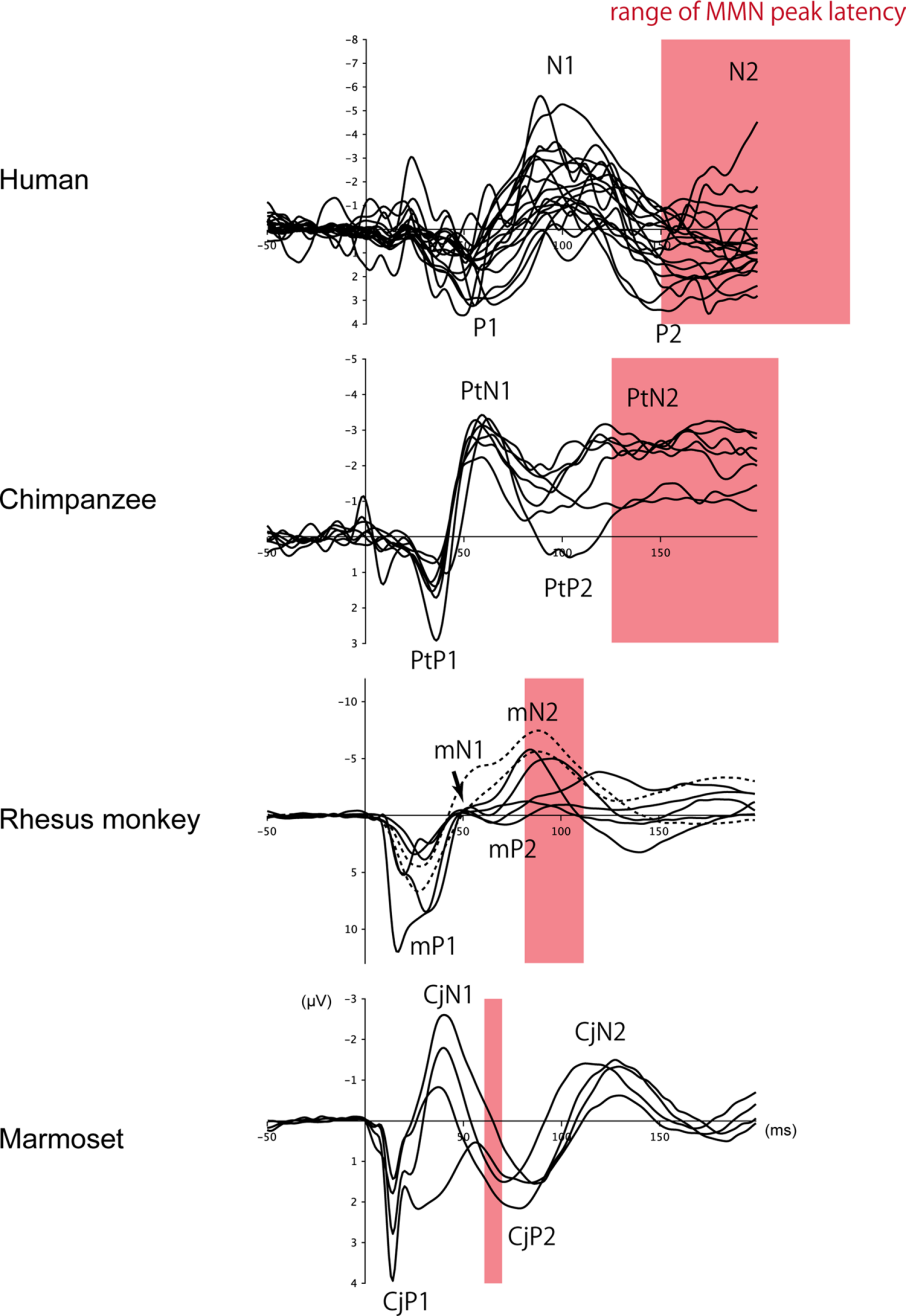
CAEP waveforms of all individual subjects (marmosets, rhesus monkeys, and humans), or all individual experiments (chimpanzee), compared with the MMN. Although there were variations in the waveforms within species or between experiments, within-species variations in the peak latencies of P1/N1 homologs were small compared to the between-species differences. Two rhesus monkeys (dotted trace) were excluded from the analysis of mN1, as their peak latency was not clearly determined. The red bands indicate the time range of the reported peak latencies of MMN in each species. Note that the MMNs occur slightly after the N1 homologs and that they increase in latency with brain size. *CAEP* cortical auditory-evoked potential, *MLR* middle latency response, *MMN* mismatch negativity.

As a limitation of the study, it is important to acknowledge that we assumed that the human and nonhuman primates’ P1s and N1s were functionally homologous to each other. Nevertheless, two lines of evidence support this assumption. First, the polarities of the CAEP components were preserved among the species. It seemed more natural to interpret the first and second prominent peaks as corresponding to P1 and N1, rather than pursue more complex interpretations. Second, and more importantly, the mismatch negativity (MMN), which reflects the detection of contextual deviance in sounds^49^, exhibits a similar evolutionary elongation of latency as the putative N1 homologs. The peak MMN latency is 150–250 ms in humans^49^, 125–210 ms in chimpanzee^14^, 80–110 ms in macaques^8,10^, and 60–70 ms in marmosets^50^, all of which are slightly longer than the latencies of their respective putative N1 homologs (Fig. 5). Therefore, it is reasonable to assume that N1, PtN1, mN1, and CjN1 reflect functionally comparable stages of auditory processing.

Another limitation was that only four species were included in the analyses. More accurate calculations of the allometric relationships would be possible, as future technical advances enable noninvasive CAEP recordings in other nonhuman primates. Moreover, future inclusions of non-primate species will provide a critical test of our hypothesis. Since primates have the highest density of cortical neurons among other mammals of similar brain size^4^, our hypothesis predicts that the CAEP latencies will be shorter in non-primate brains than in primate brains, when their brains are comparable in size. Nevertheless, notable strengths of this study were that the analysis covered a wide range of primate brain sizes, from the marmoset to the human, and that the analyses included data from a chimpanzee, which are extremely rare and valuable.

In conclusion, the enlargement of human brain size, or, more accurately, the increase in the number of cortical neurons developing over the course of primate evolution, correlated with an increase in the dwell time of auditory cortical processing in humans. This finding was logically expected, although the results were against some previous speculations that the human brain maintained a short processing time due to extensive cortical folding^1,6^, high neuronal density^4^, and thickened myelination^7^ that kept the signal conduction time short.

A longer timescale of cortical processing allows neural inputs to the cortex to be integrated over an extended time window. In the case of audition, such elongation would enable analyses of the temporal features of time-varying acoustic stimuli that are important for the perception of speech and other complex sounds. It is possible that the evolutionary prolongation of cortical processing time might have occurred not only in audition, but also in other sensory modalities, as the latencies of visual evoked potentials are also shorter in nonhuman primates than in humans^51^. These findings lead to a novel hypothesis concerning human brain evolution, such that the increase in the number of cortical neurons widened the time window of neural computation over the entire cerebral cortex. In other words, if the benefit of perceptual or cognitive enhancement due to the widened time window was sufficiently large, the number of cortical neurons would be biased to increase during evolution despite the cost of longer processing time. The hypothesis that higher brain functions are supported by longer timescales of processing is consistent with the previous findings that the timescale of spontaneous firing at the level of single neurons is longer in higher association cortices compared to sensory cortices in the non-human primate brain^52,53^, and that higher-tier cortical areas shows extended timescales of neural processing in the human brain^54^. Further clarification of differences between species in how the brain handles time may provide a key to understanding the evolution of human intelligence.

## Materials and methods

### Experimental design

The raw data obtained in several published^9,11–14^ and unpublished experiments from our group were reanalyzed for the purpose of this study. Methods that were common to all experiments were as follows: EEGs were measured noninvasively from the scalp, subjects listened to the sounds passively without performing a behavioral task; and the sound pressure levels were medium to loud. There were also minor differences, such as the experiment site, EEG amplifier, and specific stimulus parameters, which are described below. The study is reported in accordance with the recommendations in the ARRIVE guidelines (https://arriveguidelines.org) for all nonhuman primate species.

### Marmoset EEG recording

Three experiments were conducted on four adult common marmoset monkeys, consisting of three males (subject names: Cj399, Cj419, Cj459; body weight: 260–330 g; 5–6 years old) and one female (Cj368, 320 g, 7 years old). Not all subjects were used in all of the experiments. The details of animal preparation and EEG recording method are described elsewhere^11^. Briefly, the monkey sat in a primate chair and free movement of the head was restricted by a thermoplastic mask that was adjusted to the size and shape of each animal. Silver dish electrodes with a diameter of 4 mm (Unique Medical, Tokyo, Japan) were placed on the shaved scalp according to the International ten-twenty system. The electrodes were fixed using adhesives, and electrode gel was applied to lower the impedance to below 5 kΩ. All sounds were presented at an intensity of 65–70 dB SPL.

In Experiment 1^11^, the auditory stimulus was a pure tone (1500 Hz) with a duration of 300 ms (6 ms linear rise/fall times). The sound was presented at two different stimulus rates: fast and slow. In the fast-rate condition, the stimulus-onset asynchrony (SOA; the time interval between the onsets of two consecutive sounds) was 330 ms, and in the slow-rate condition, the SOA was 1130 ms. Only the slow-rate condition was analyzed because the CAEPs were not clearly elicited in the fast-rate condition. All four monkeys were used in this experiment. A total of 2000–4000 trials were presented depending on the subject, and 97.9–99.2% of those were accepted for analysis after artifact rejection (see below).

In Experiment 2 (unpublished), the auditory stimulus was a pure tone (1500 Hz) that had various durations in the range of 2–150 ms (1 ms linear rise/fall times). Only the CAEPs to sounds with a duration of 50 ms or longer were analyzed, so that the stimulus condition matched that of the human version of this experiment (see below). The sound was presented with an inter-trial silent interval of 300–400 ms. Other aspects of the experiment were the same as in Experiment 1. All four monkeys were used in this experiment. A total of 2800–7000 stimuli were presented depending on the subject, and 96.3–99.5% of those were accepted for analysis.

In Experiment 3 (unpublished), five stimuli of different frequencies (500, 750, 1125, 1688, and 2531 Hz) were presented randomly and equiprobably in sequence. The sound duration was 200 ms, and the SOA was 600 ms. The CAEPs for all frequency conditions were pooled for analysis. Two monkeys (Cj399 and Cj459) were used in this experiment. A total of 2000 trials were presented for each subject, and 98.6–99.9% of those were accepted for analysis.

After obtaining the CAEPs, the latencies of CjP1 and CjN1 were measured at the Cz electrode for each experiment and each subject. Next, the latencies were averaged across the experiments for each subject. Finally, the measurements were averaged over all subjects to obtain the CjP1 and CjN1 latencies for the marmosets. Figure 1 shows the grand-averaged CAEPs of all the experiments and all the subjects. The waveforms of the individual subjects are shown in Fig. 5.

Presentation software (Neurobehavioral Systems, Berkeley, CA) and Sound Blaster audio hardware (Creative Technology, Jurong East, Singapore) were used for stimulus presentation. Sound playback latencies with respect to the trigger signal were measured at the point of signal output from the computer, and these were corrected in post-processing. There was also a small delay of ~ 2 ms that reflected the traveling time of the sound wave from the loudspeaker to the subject (0.6 m); this was also corrected in post-processing. EEG was recorded using BrainAmp MR plus (Brain Products, Munich, Germany).

All marmoset experiments were conducted at the Primate Research Institute of Kyoto University (Aichi, Japan) and adhered to the Guide for the Care and Use of Laboratory Animals of the National Research Council (1996) and the Guide for Care and Use of Laboratory Primates published by the Primate Research Institute of Kyoto University (2010). The experiment adhered to the legal requirements that apply to animal research conducted in Japan and was approved by Kyoto University and Niigata University.

### Macaque EEG recording

Four experiments were conducted on seven adult or young adult rhesus monkeys, consisting of five males (subject names: Mm1979, Mn1961, Mm1963, Mm1866, and Mm1789; body weight: 4.1–7.5 kg; 3–10 years old) and two females (Mm1892 and Mm2033; body weight, 4.5–6.2 kg, 4–7 years old). Not all subjects were used in all of the experiments. The details of the animal preparation and EEG recording method are described elsewhere^12,13^. Briefly, the monkey sat in a primate chair and its head was fixed by using bars or thermoplastic masks that were adjusted to the size and shape of each animal. Silver electrodes for human sleep recording (NE-136A, Nihon Kohden, Japan) were placed on the shaved scalp according to the International ten-twenty system. The electrodes were fixed using gauze soaked with collodion, and electrode gel was applied between the electrode and the scalp to lower the impedance to below 5 kΩ. All sounds were presented at an intensity of 65–70 dB SPL, except in Experiment 4.

Experiment 1 was a reanalysis of our previously published data^12^, in which a single pure tone (1500 Hz) was presented repeatedly. The stimulation conditions were identical to those of Experiment 1 in the marmoset study, except that the SOA was 300 ms and 1100 ms for the fast- and slow-rate conditions, respectively. Only the slow-rate condition was analyzed, as in the marmoset experiment. Three monkeys (Mm1892, Mm1963, and Mm1789) were used in this experiment. A total of 923–7039 stimuli were presented depending on the subject, and 88.0–94.1% of those were accepted for analysis.

Experiment 2 was a reanalysis of the data obtained in a previous study^13^, in which a pure-tone stimulus (1500 Hz) of varying durations (2–100 ms) was presented, similar to Experiment 2 in marmosets. Only CAEPs with a duration of 50 ms or longer were analyzed, as in the marmoset study. Six monkeys (Mm1979, Mm1892, Mm1961, Mm1963, Mm2033, and Mm1866) were used in the original experiment (*13*), but another subject (Cj2033) was added for the present study. A total of 8250–12,000 stimuli were presented depending on the subject, and 85.0–94.7% of those were accepted for analysis.

Experiment 3 (unpublished) was similar to that of Experiment 3 in marmosets. Five stimuli of different frequencies (667, 1000, 1500, 2250, and 3375 Hz) were presented randomly and equiprobably in sequence. The sound duration was 300 ms, and the SOA was 570 ms. All frequency conditions were pooled. Two monkeys (Mm1892 and Mm1866) were used in this experiment. A total of 4049–7990 stimuli was presented depending on the subject, and 76.9–84.7% of those were accepted for analysis.

Experiment 4 (unpublished) was an MMN experiment, in which a click stimulus (0.1 ms duration) was presented at two levels of intensity in a random order: (95 dB LC_peak_) or soft (75 dB LC_peak_). In the standard soft condition, the probability of stimulus presentation was 0.8 and 0.2 for the soft and loud clicks, respectively; the probabilities were reversed in the standard loud condition. Only the CAEPs to the standard stimuli were analyzed, indistinguishing the soft and loud standards. A total of 5435–5440 standard stimuli were presented depending on the subject, and 79.2–91.1% of those were accepted for analysis.

After obtaining the CAEPs, the mP1 and mN1 latencies were measured at Cz for each experiment and for each subject. Next, the latencies were averaged across the experiments for each subject. Finally, the measurements were averaged over all subjects to obtain the mP1 and mN1 latencies for rhesus monkeys. Subjects Mm1961 and Mm1866 were excluded from the analysis of mN1 because they did not elicit a clear mN1. The waveform in Fig. 1 was obtained by averaging the CAEPs across all experiments and all subjects, excluding those that did not elicit a clear mN1. Individual waveforms of all subjects, including Mm1961 and Mm1866, are shown in Fig. 5.

Presentation software (Neurobehavioral Systems, Berkeley, CA) and Sound Blaster audio hardware (Creative Technology, Jurong East, Singapore) were used for stimulus presentation. Sound playback latencies with respect to the trigger signal were measured at the point of signal output from the computer, and these were corrected in post-processing. Even after this correction, there remained a small delay of ~ 2 ms that reflected the traveling time of the sound wave from the loudspeaker to the subject (0.6 m), which was also corrected in post-processing. EEG was recorded using BrainAmp MR plus (Brain Products, Munich, Germany).

All rhesus monkey experiments were conducted at the Primate Research Institute of Kyoto University (Aichi, Japan) and adhered to the Guide for the Care and Use of Laboratory Animals of the National Research Council (1996) and the Guide for Care and Use of Laboratory Primates published by the Primate Research Institute of Kyoto University (2010). The experiment adhered to the legal requirements for the conduct of animal research in Japan and was approved by Kyoto University and Niigata University.

### Chimpanzee EEG recording

Three experiments were performed on a single nine-year-old chimpanzee named Mizuki. A larger number of chimpanzees would have made the findings more robust. However, such opportunity is extremely rare, and the inclusion of the chimpanzee data is a strength of this study rather than a limitation. Details of animal preparation and the EEG recording method are described elsewhere^9,14^. Briefly, the method of electrode application was similar to that for macaque monkeys, except that the animal was not physically restrained from free body and head movements. All sounds were presented at an intensity of 80 dB HL. Seven CAEPs were obtained from the chimpanzee, as described below, and the waveform in Fig. 1 shows their grand average.

Experiment 1 was a reanalysis of a previously published MMN experiment^14^, in which a pure-tone stimulus of one frequency was presented as the standard stimulus (*p* = 0.8) and another pure-tone stimulus of a different frequency as the deviant stimulus (*p* = 0.2). Only the CAEPs to the standard stimulus were analyzed. The sound duration was 100 ms, and the SOA was 700 ms. The stimulus frequency varied across four sessions (500 Hz vs. 1500 Hz, 500 Hz vs. 2000 Hz, 1500 Hz vs. 500 Hz, 2000 Hz vs. 500 Hz), and the different sessions were analyzed separately; that is, four independent CAEPs were obtained to measure the PtP1 and PtN1 latencies. A total of 1460–1605 standard stimuli were presented depending on the session, and 96.2–97.7% of those were accepted for analysis.

Experiment 2 was a standard CAEP experiment, in which a 500 Hz pure tone (duration 100 ms) was presented repeatedly with a fixed SOA of 700 ms. A total of 1615 trials was presented, and 96.8% of those were accepted for analysis.

In Experiment 3, the auditory stimulus was the subject’s own name (Mizuki) that was uttered by a human in an angry or a friendly voice. This experiment was a variation of another study that recorded CAEP to the subject’s own name^9^. The name stimuli were presented at a probability of *p* = 0.1 each, and a pure-tone stimulus (500 Hz, 100 ms duration) was presented at a probability of *p* = 0.8. Two CAEPs were obtained one for the name stimuli without distinguishing the voices, and the other for the pure-tone stimulus. A total of 3128 trials was presented for the name stimuli, and 97.0% of the trials were accepted for analysis. A total of 12,458 trials was presented for the pure tone, and 97.0% of those were accepted for analysis. Although the name stimulus was acoustically more complex than pure-tones, the latencies of P1 and N1 were resilient to the stimulus difference, as shown in Fig. 5.

After obtaining the CAEPs, the PtP1 and PtN1 latencies were measured at Cz for each experiment. Next, the latencies were averaged across experiments to obtain the PtP1 and PTN1 latencies for the chimpanzee. Individual waveforms of all experiments are shown in Fig. 5, and the grand average is shown in Fig. 1.

The stimulus delivery hardware (STIM2, Neuroscan, Inc.) was configured to have no sound playback latency with respect to the trigger signal. There was a delay of ~ 6 ms that reflected the traveling time of the sound wave from the loudspeaker to the subject (2 m), which was corrected in post-processing. The EEG signals were obtained using a NuAmp-40 amplifier (NeuroScan Inc., El Paso, USA).

All chimpanzee experiments were conducted at the Great Ape Research Institute, Hayashibara Biochemical Laboratories, Inc. (Okayama, Japan). The experiment adhered to the legal requirements of Japan and the research protocol was approved by the Research Ethics Committee of the Hayashibara Great Ape Research Institute.

### Human EEG recording

Human EEG data were obtained as an integrated part of the corresponding nonhuman primate experiments described above for species comparisons. Therefore, the stimuli and recording conditions were matched as closely as possible within those experiments.

Experiment 1 was a human version of the Experiment 2 in rhesus monkeys, the details of which are described above and elsewhere^13^. Twelve subjects (two males and ten females, aged 18–23 years) participated in the study. Pure-tone stimuli (1500 Hz) of varying durations (2–200 ms) were presented. However, only the CAEPs to sounds with a duration of 50 ms or longer were analyzed, because the latencies of P1 and N1 were clearly longer for shorter sounds; see Fig. 1 in^13^. A total of 1794–1800 stimuli were presented depending on the subject, and 97.4–99.9% of those were accepted for analysis.

The Presentation software (Neurobehavioral Systems, Berkeley, CA) and Sound Blaster audio hardware (Creative Technology, Jurong East, Singapore) were used for stimulus presentation in Experiment 1. Sound playback latencies with respect to the trigger signal were measured at the point of signal output from the computer, and these were corrected in post-processing. In addition, there was a small delay of ~ 4 ms, which reflected the traveling time of the sound wave from the loudspeaker to the subject (1.2 m); this was also corrected in post-processing. The EEG was recorded using a BrainAmp DC (Brain Products, Munich, Germany).

The experiment was conducted at Niigata University after the Internal Review Board of the University of Niigata approved the study. All experiments were performed in accordance with the principles of the Declaration of Helsinki. Informed consent was obtained from all participants.

Experiment 2 was a human version of the chimpanzee’s Experiment 2, and the stimuli were identical. Six subjects (two males and four females, aged 23–53 years) participated in the study. A total of 100–1000 trials was presented depending on the subject, and 97.4–100% of those were accepted for analysis. The EEG signals were obtained using NuAmp-40 (NeuroScan Inc., El Paso, USA).

The stimulus delivery hardware (STIM2, Neuroscan, Inc.) was configured to have no sound playback latency with respect to the trigger signal. There was a delay of ~ 6 ms that reflected the traveling time of the sound wave from the loudspeaker to the subject (2 m), which was corrected in post-processing.

The experiment was conducted at the Great Ape Research Institute, Hayashibara Biochemical Laboratories, Inc. (Okayama, Japan). The research protocol was approved by the Research Ethics Committee of the Hayashibara Great Ape Research Institute. Informed consent was obtained from all participants.

### Data analyses

The EEG data were analyzed similarly for all experiments in all species to obtain the CAEPs, utilizing the EEGLAB software^55^ with the ERPLAB extension^56^. Only the Fz, Cz, Pz, and ear channels were used, where Fz, Cz, and Pz refer to the electrode positions as specified by the 10–20 system. The data were first bandpass filtered (1–100 Hz), and re-referenced to the average of both ears in the marmoset, macaque, and human, or to the left ear in the chimpanzee (as data were not recorded from the right ear in the chimpanzee). The continuous EEG data were then segmented, time locked to the onset of the stimulus (− 50 to 200 ms), corrected for baseline by subtracting the pre-stimulus period average, screened for artifacts, and accordingly rejected if they contained an artifact of ± 100 μV relative to the baseline. One CAEP was obtained for each experimental condition for each subject. These waveforms were used for two further analyses: inspection of waveform morphology and latency measurement.

For the inspection of waveforms, the CAEPs were averaged over experimental conditions for each subject, and then over subjects within species to obtain a grand-averaged CAEP for each species (Fig. 1).

For the latency measurement, the latencies were recorded, where an inflection point of the CAEP waveform represented the local minimum (N1) or the local maximum (P1) within the respective time windows that were defined by inspecting the grand-average waveforms (Fig. 1). The time window for P1 was defined as 0–100 ms in humans and 0–50 ms in the other species, as measured at Cz. The time window for N1 was 50–150 ms in humans and 30–80 ms in the other species, as measured at Cz. Two rhesus monkeys were excluded from the analysis of mN1, as the CAEP amplitude decreased monotonically and showed no inflection point within the mN1 time window (Fig. 5).

For the allometric analysis of P1/N1 latencies and brain size (Fig. 3), the latencies were averaged over subjects to obtain a single latency measure for each species, so that the number of cases matched between the species for equal weighting in regression. Then, the data were fitted by linear regression, using the equivalent brain radius (*R*_eq_) or the number of neurons (*N*) as the independent variable, using the CURVEFIT function of IBM SPSS Statistics version 25 (IBM, Armonk, NY, United States).

## Data Availability

The data that support the findings of this study are available from the corresponding author upon reasonable request.
